# Could Time Detect a Faking-Good Attitude? A Study With the MMPI-2-RF

**DOI:** 10.3389/fpsyg.2018.01064

**Published:** 2018-07-25

**Authors:** Paolo Roma, Maria C. Verrocchio, Cristina Mazza, Daniela Marchetti, Franco Burla, Maria E. Cinti, Stefano Ferracuti

**Affiliations:** ^1^Department of Human Neuroscience, Sapienza University of Rome, Rome, Italy; ^2^Department of Psychological, Health, and Territorial Sciences, University “G. d’Annunzio”, Chieti-Pescara, Chieti, Italy

**Keywords:** MMPI-2-RF, faking-good, speed, response latency, self-report, malingering

## Abstract

**Background and Purpose:** Research on the relationship between response latency (RL) and faking in self-administered testing scenarios have generated contradictory findings. We explored this relationship further, aiming to add further insight into the reliability of self-report measures. We compared RLs and T-scores on the MMPI-2-RF (validity and restructured clinical [RC] scales) in four experimental groups. Our hypotheses were that: the Fake-Good Speeded group would obtain a different completion time; show higher RLs than the Honesty Speeded Group in the validity scales; show higher T-Scores in the L-r and K-r scales and lower T-scores in the F-r and RC scales; and show higher levels of tension and fatigue. Finally, the impact of the speeded condition in malingering was assessed.

**Materials and Methods:** The sample was comprised of 135 subjects (*M* = 26.64; *SD* = 1.88 years old), all of whom were graduates (having completed at least 17 years of instruction), male, and Caucasian. Subjects were randomly assigned to four groups: Honesty Speeded, Fake-Good Speeded, Honesty Un-Speeded, and Fake-Good Un-Speeded. A software version of the MMPI-2-RF and Visual Analog Scale (VAS) were administered. To test the hypotheses, MANOVAs and binomial logistic regressions were run.

**Results:** Significant differences were found between the four groups, and particularly between the Honest and Fake-Good groups in terms of test completion time and the L-r and K-r scales. The speeded condition increased T-scores in the L-r and K-r scales but decreased T-scores in some of the RC scales. The Fake groups also scored higher on the VAS Tension subscale. Completion times for the first and second parts of the MMPI-2-RF and T-scores for the K-r scale seemed to predict malingering.

**Conclusion:** The speeded condition seemed to bring out the malingerers. Limitations include the sample size and gender bias.

## Introduction

A common concern for those using self-report inventories of personality and psychopathology is the susceptibility of such inventories to malingering or faking (Anastasi, 1988; Holden et al., 1992). Ziegler et al. (2012) defined faking as an intentional and deliberate behavior that helps an individual achieve personal goals. Specifically, *fake-good behavior* involves presenting the self in a more positive manner, relative to honest self-evaluation (Maricuţoiu and Sârbescu, 2016). In any assessment setting, a subject completing a personality inventory can answer truthfully or not, according to his or her goal. For this reason, detection of malingering represents an area of considerable interest for researchers of individual differences (Holden et al., 2001). Over the past years, psychologists have searched for methods to identify the occurrence of this phenomenon (Fluckinger et al., 2008).

In the 1970s, Dunn et al. (1972) suggested that *response latency* (RL; i.e., the amount of time elapsed between an item’s presentation and a subject’s response) could be used to detect dissimulation tendencies. Beginning in the 1990s, RL was proposed more insistently as an additional method of testing the validity of the Minnesota Multiphasic Personality Inventory (MMPI; Hathaway and McKinley, 1951), together with the MMPI’s own validity scales (L-r, F-r, and K-r).

Nevertheless, over the decades, divergent perspectives regarding RL and faking have emerged in the literature, and empirical research has produced mixed findings. According to the *semantic evaluation perspective* (Hsu et al., 1989), shorter RL is associated with higher scores on social desirability scales, because it is easier to evaluate the meaning of an item than to evaluate that item according to autobiographic information, which involves recalling episodes to direct the answer. More specifically, Dunn et al. (1972) found, in administering the MMPI, that participants in faking conditions had shorter RLs relative to participants in honesty conditions. Hsu et al. (1989), referring to the theories of response process proposed by Nowakowska (1970), Rogers (1971, 1977), and Kuncel (1973), studied the RL in the subtle-obvious scales of the MMPI on a sample of 100 undergraduate students who were instructed to fake-bad or fake-good, with or without an incentive. The results indicated that RL was shorter in the fake condition and that RL had incremental validity in detecting both faking-good and faking-bad. This finding is supported by the theory that responding to MMPI items with the intent to dissemble involves accessing a less elaborate information schema or network (Brunetti et al., 1998).

Several researchers have proposed theories and shown empirical results that diverge from the idea that faking speeds the processing of personality test items. Authors who support the *self-schema model* (McDaniel and Timm, 1990; Holden and Kroner, 1992; Holden et al., 1992; Walczyk et al., 2003, 2005; Foerster et al., 2013) argue that faking is a complex process that, relative to honest answering, requires extra cognitive processing and editing. Maricuţoiu and Sârbescu (2016) assumed that “honest respondents answer consistently with their self-schemas, while dishonest respondents decide not to provide self-schematic information, after an evaluation of schematic information” (p. 2). Vasilopoulos et al. (2000) stated that fakers must reflect and, in turn, keep real information in memory, and they must inhibit and replace this real information with fake information taken from the target’s ideal schema. This schema is hypothesized and, for this reason, more complex and not immediately available for recall; thus, it takes longer for faking respondents to provide an answer (see also DePaulo et al., 2003). Honest respondents, in contrast, are able to respond automatically and spontaneously, and thus they use fewer cognitive processes than malingerers and their RL is correspondingly shorter. According to these authors, fakers’ larger RLs are due to higher levels of arousal, generated by their fear of being detected.

An interesting variant of the *self-schema model* was introduced by Holden (1995). The author found shorter RLs when items were congruent to the faking scheme: if subjects were asked to describe themselves in the best possible way (i.e., comply with a *fake-good scheme*), they registered shorter RLs on items describing socially desirable behaviors. A reverse pattern was observed for items incongruent with the scheme. Similar results were obtained by Holden and Lambert (2015) using the NEO-PF inventory (Costa and McCrae, 1992) and by Brunetti et al. (1998) using MMPI-2 (Hathaway and McKinley, 1989; Butcher et al., 2001). These authors showed that subjects required significantly more time to respond to items that were incongruent with their response set.

Some studies on RL have also evaluated the *pressure of time* effect on faking behavior with personality inventories. Khorramdel and Kubinger (2006) found that faking when responding to dichotomous items was accentuated under time pressure, and thus a time limitation may drive people to increase their faking behavior in the direction required by the instructions (data also reported by Holden et al., 2001). Shalvi et al. (2013) showed that subjects lie more frequently when they have little time to reflect; when they have more time at their disposal, they reflect more deeply on their response and moderate the simulation. Time pressure, therefore, seems an important factor in faking behavior.

While a theoretical basis may exist for the use of latencies in faking detection, previous research on the association of RL with faking has yielded mixed results and, recently, contradictory findings (fakers are faster, Maricuţoiu and Sârbescu, 2016; fakers are slower, Van Hooft and Born, 2012). Therefore, in the current research, we were interested in increasing the understanding of RL by merging it with a time pressure condition to determine whether the combination of these factors can help detent faking behavior.

Dividing our sample into an honest group (H) and a group instructed to fake-good (FG), we used a common self-administered inventory of personality and psychopathology, together with two conditions of time (speeded [S] and un-speeded [U]), to test the following hypotheses:

H1: There would be significant differences in the protocol’s total completion time. Analysis of these differences could increase our knowledge of fakers’ test compiling attitudes, in both unrestricted (U) and speeded (S) time conditions.
H2: There would be significant differences in completion times for the protocol’s parts, both within and between groups. In the H groups, we expected a fatigue effect, resulting in progressively higher completion times. In the FG groups, we expected both a fatigue effect and a learning effect, due to the difficulty of learning the FG response model. Studying the partial time responses within groups and the differences across groups in each section could provide a deeper understanding of the information processing of honest and faking respondents.
H3: There would be significant differences in RLs between groups in self-presentation measures on the self-administered inventory. We investigated the RLs of the self-presentation scales, in particular, since these were thought to be useful for differentiating between H and F respondents.
H4: There would be differences between groups in self-presentation scores, with FG groups reporting higher values in positive self-presentation, lower scores in negative self-presentation, and lower values in psychopathology, relative to H groups.
H5: There would be differences in tension and fatigue levels between the H and FG groups. We wanted to study the influence of these variables on RL and inventory scores.
H6: The RLs identified in H1, H2, and H3 would be effective for predicting faking behavior.

As introduced in the hypotheses, we chose to restrict the analysis to a comparison between H and FG schemes. We chose FG for this study as it is more common than the fake-bad scheme, and thus the application of results would be more extensive. In other words, it is more likely that a situation will drive a subject to exhibit fake-good behaviors (e.g., during personnel selection or qualifying examinations) than fake-bad behaviors. Regarding the measure used, we chose the Minnesota Multiphasic Personality Inventory-2-Restructured Form (MMPI-2-RF; Ben-Porath and Tellegen, 2008), as it has been extensively used in clinical (see, e.g., Anderson et al., 2015) and selection settings (see, e.g., Tarescavage et al., 2015), but not yet used in latency studies. Furthermore, to the best of our knowledge, no prior study has addressed RL and MMPI scores under time pressure conditions.

## Materials and Methods

### Participants

Subjects were 140 young adult volunteers who participated in the study for a small reward (European breakfast in a cafe). To limit confounding variables, we recruited only subjects who were aged 25–30 years (*M* = 26.64; *SD* = 1.88 years), male, Caucasian, graduates (having completed at least 17 years of education), and non-psychology graduates (i.e., those who had not attended the faculty of psychology). Subjects participated in the trial in the morning and were randomly assigned to one of four instruction groups. Six subjects were excluded from data analysis for one or more of the following reasons: (a) failure to follow instructions as assessed by the final request (*n* = 2), (b) one or more changes in answers (*n* = 3), or (c) too brief a latency in one or more responses (*n* = 1, 3000 m/s). The remaining 135 subjects composed the research group. No statistically significant differences were observed on age or level of education. Data were collected over a period of 2 months, from October to November 2017.

### Materials

#### MMP-2-RF

The full Italian version of the MMPI-2-RF (Sirigatti and Faravelli, 2012) was used. The MMPI-2-RF (Ben-Porath et al., 2008/2011) is a 51-scale measure of personality and psychopathology with 338 items, selected from the 567 of the MMPI-2 (Tellegen et al., 2003; Ben-Porath and Tellegen, 2008). In particular, this study used the T-scores of the three principal validity scales (L-r, F-r, and K-r) and the nine restructured clinical (RC) scales (to assess H4). We chose these scales as they represent the test’s core evaluative measures and because our sample was not sufficiently large to guarantee a reliable analysis of all 51 scales (see **Table 1** for a brief description of the 12 selected scales). For our study, we added a Total scale, which was the sum of the *T*-scores of each of the nine RC scales. This Total scale was similar to the MMPI-2’s “total elevation of protocol.” T-scores (*M* = 50, *SD* = 10) are the traditional unit of measurement in the MMPI-2 (Tellegen and Ben-Porath, 1992), and they are also used in the MMPI-2-RF. The *T*-scores classification is: 45–54 (*average*), 55–69 (*slightly high*), 60–64 (*moderately high*), 65–69 (*high*), and 70–79 (*very high*) (Butcher et al., 2001).

**Table 1:** Selected MMPI-2-RF scales.

Scale	Title	What is measured
L-r	Uncommon Virtues	Infrequent and therefore improbable virtues
F-r	Infrequent Responses	Infrequent symptomatology
K-r	Adjustment Validity	Adaptation to life
RCd	Demoralization	Unhappiness and dissatisfaction with life
RC1	Somatic Complaints	Pattern of somatic complaints
RC2	Low Positive Emotions	Depressive symptoms
RC3	Cynicism	Negative view of human nature
RC4	Antisocial Behavior	Antisocial behavior and related family conflict
RC6	Ideas of Persecution	Persecutory beliefs
RC7	Dysfunctional Negative Emotions	Various negative emotional experiences
RC8	Aberrant Experiences	Thinking disorders
RC9	Hypomanic Activation	High level of activation and engagement

We also assessed the completion time for the entire protocol (to assess H1) and the completion times for each of the three consecutive parts, which were composed of a similar number of items (112 for the first part, 112 for the second, and 114 for the third, in order to assess H2); and the RL of the three principal validity scales (to assess H3).

#### Visual Analog Scale (VAS)

The VAS is a simple technique for measuring subjective experience (McCormack et al., 1988). It consists of a 10 cm line segment with two extreme polarities. Subjects must place a single mark on the line to indicate the current level of their experience (0 = the best possible condition, 10 = the worst possible condition). In our experiment, VAS was used to assess subjects’ levels of tension (anxiety) (VAS-T) and fatigue (VAS-F), both before (T0) and after (T1) the MMPI-2-RF evaluation. The difference between VAS at T1 and T0 was used to understand changes in subjects’ levels of tension and fatigue.

#### Software Application

We implemented an application for Android devices, with all 338 items loaded onto the platform. Participants used their dominant hand (126 right-handed, 9 left-handed) to press the virtual key F (false, on the bottom left) or V (true, on the bottom right) on the application. Following this response, the next item would appear immediately on the screen. At the top of the screen a red virtual button would offer subjects the possibility to return to the previous question. The program simultaneously recorded subjects’ responses (V or F) and RL (measuring the time between the appearance of an item to the subject’s tap of the virtual key) for each item. The same device was used for all uses of the application, and the application was stored on the device (rather than accessed online), so that Internet speed would not influence RL.

### Research Design

A 2 × 2 between-subjects design was used. The two manipulated factors were instruction (H vs. FG) and time pressure (U vs. S). Participants were randomly assigned to one of four experimental groups of 35 persons: H/U, FG/U, H/S, and FG/S. The four instructions were:

(1) H/U: “We are interested in some characteristics of your personality. We want you to take this test in a totally sincere fashion. After reading each item you should take all the time you need to respond in the best way.”
(2) FG/U: “We are interested in some characteristics of your personality. Imagine you are applying for a desired job. In this situation, it would be to your advantage to appear as if you were completely normal and psychologically healthy. Stated differently, we want you to take this test and deliberately fake good. Pay attention, because the questionnaire contains features designed to detect faking, and your intent is to respond in a way that your deception cannot be detected. After reading each item you should take all the time you need to respond in the best way, according to this instruction.”
(3) H/S: “We are interested in some characteristics of your personality. We want you to take this test in a totally honest fashion. After reading each item you should respond as quickly as possible. Short response time is an important factor in this test.”
(4) FG/S: “We are interested in some characteristics of your personality. Imagine you are applying for a desired job. In this situation it would be to your advantage to appear as if you were completely normal and psychologically healthy. Stated differently, we want you to take this test and deliberately fake good. Pay attention, because the questionnaire contains features designed to detect faking, and your intent is to respond in a way that your deception cannot be detected. After reading each item you should respond as quickly as possible. A short response time will enable you to stand out positively from other candidates.”

### Procedures

The subject, placed in front of a device on a 70 cm high desk with an adjustable height chair set at a distance of about 40 cm (with the back straight on the chair), received the following information and questions: (a) an explanation of the research and procedure, (b) a consent form, (c) a demographic questionnaire, (d) the T0 VAS (on white paper), (e) a brief introduction to the platform, (f) 10 training questions on the device, (g) 10 neutral questions (for which the average response time was collected), (h) instructions for the task, (i) the MMPI-2-RF test, (j) the T1 VAS (on white paper), and (k) a final check of their understanding of the instructions, as follows: after the trial, subjects performed two tasks designed to test their understanding: (1) write briefly on the card next to the device the initial instructions, and (2) write whether they thought they had followed the instructions when completing the protocol. Two participants proved not to have understood the task (1) and one subject declared not to have followed instructions during the test (2).

### Statistical Analyses

In order to assess potentially noisy variables between the four groups (such as motor speed and reading speed) at the beginning, we ran an ANOVA to test for significant differences in RL in the 10 neutral questions (procedure point g).

Multivariate analyses of variance (MANOVAs) were run with the two attitudes toward the test conditions (H vs. FG) and the two speed groups (U vs. S) used as independent variables. Times of fulfillment, RL in the selected scale, T-scores, and VAS measures served as the dependent measures. Scheffé’s (1959) method was used to assess *post hoc* pair differences (*p* < 0.05). Effect size was calculated using partial eta squared. Values of 0.02, 0.13, and 0.26 were considered indicative of small, medium, and large effects, respectively (Pierce et al., 2004). Binomial logistic regression was run to evaluate the discriminatory power of the variables related to time (dependent variable), with respect to the H condition (fixed factor).

## Results

The ANOVA showed a non-significant difference between groups [*F*(3,131) = 1.585; *p* = 196] on RL in the 10 neutral questions. No differences between groups were found on verbal ability or motor speed. We decided, however, to run MANCOVAs with the 10 neutral questions as covariates. As no significant covariate effect was found, we decided not to include the neutral questions in the final analysis.

### Variables Related to Time

In the seven variables related to completion time and RL, the MANOVAs revealed a significant effect of honesty [Wilks’ lambda F(3,125) = 91.503; p < 0.001; $\eta_{\text{p}}^{2}$ = 0.813], speed [Wilks’ lambda F(3,125) = 125.583; p < 0.001; $\eta_{\text{p}}^{2}$ = 0.853], and the interaction of honesty and time [Wilks’ lambda F(3,128) = 8.472; p < 0.001; $\eta_{\text{p}}^{2}$ = 0.287]. **Table 2** shows the descriptive values of the four groups for the protocol and validity scale fulfillment times.

**Table 2:** Means and SDs of the four experimental groups for MMPI-2-RF completion time and RL in the three validity scales, with *post hoc* test results.

	H/U (*n* = 35) *M* (*SD*)	FG/U (*n* = 33) *M* (*SD*)	H/S (*n* = 33) *M* (*SD*)	FG/S (*n* = 34) *M* (*SD*)
**Completion time (min)**		
Items 1–112	7.46 (0.99) A	11.56 (1.30) B	5.68 (1.06) C	8.12 (1.27) A
Items 113–224	10.57 (1.19) A	11.07 (1.25) A	7.21 (0.65) B	8.76 (1.12) C
Items 225– 338	12.64 (1.23) A	13.87 (1.67) B	9.74 (1.31) C	11.12 (2.32) D
Total time	30.67 (1.76) A	36.49 (2.19) B	22.64 (1.82) C	28.01 (1.78) D
**Validity scales completion time (sec.)**				
L-r	3.43 (1.14) A	5.52 (1.69) B	2.47 (0.40) C	4.52 (0.82) D
F-r	3.71 (1.52) A, B	4.47 (2.48) A	3.23 (1.56) C	2.75 (0.58) B, C
K-r	4.86 (1.36) B	6.23 (1.58) A	3.83 (0.38) C	6.28 (0.81) A

*H, honest; U, un-speeded; FG, faking-good; S, speeded; L-r, Lie scale; K-r, Correction scale; F-r, Frequencies scale. For each line, different letters indicate a significant difference between columns.*

Regarding total completion time, the H/S group was fastest, followed by the FG/S, H/U, and FG/U groups. Therefore, both FG groups were slower than the H groups in the same speed condition (U: H = 30.67 min vs. FG = 36.49 min; S: H = 22.64 min vs. FG = 28.01 min). Contrasting the three partial completion times between the four groups, the results showed that the S condition—in both the H and FG groups—always reduced execution time by 2 or 3 min per section, relative to the U condition. The FG/S group was slower than the H/S group in completing all three sections. In the U condition, H was faster than FG in the first and third sections, while both conditions showed equal means in the second section.

Analyzing within-group differences, subjects of the H groups (in both time conditions) showed progressive and significant increases in completion times from the first to the third section. FG groups showed a different pattern, with quite similar completion times for the first two sections and a shorter time for the third section.

**FIGURE 1:**
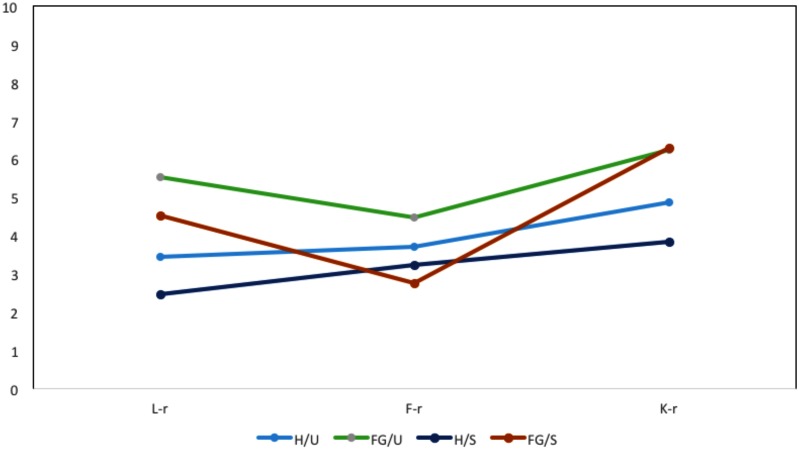
Response latency (RL) (in ms) in the three MMPI-2-RF validity scales.

In the L-r scale, H groups were faster (first H/S, then H/U) than FG groups. FG groups showed a significant difference of about 1 second between FG/S (faster) and FG/U. In the K-r scale, FG groups showed the same RL and were slower than H groups, for whom the H/S group showed the fastest times. In the F-r scale, groups diverged in the S condition (with H/S faster than H/U and FG/S faster than FG/U), though the average value of the H/U group did not significantly differ from that of the FG/S group (see **Figure 1**).

### T-Scores in MMPI-2-RF

In the 12 variables related to *T*-scores, MANOVAs revealed a significant effect of honesty [Wilks’ lambda *F*(3,125) = 27.308; *p* < 0.001; $\eta_{\text{p}}^{2}$ = 0.732] and speed [Wilks’ lambda *F*(3,125) = 3.209; *p* < 0.001; $\eta_{\text{p}}^{2}$ = 0.243], and a non-significant effect of the interaction between honesty and time [Wilks’ lambda *F*(3,128) = 1.726; *p* = 0.069; *d* = 0.147]. **Table 3** reports the descriptive *T*-scores for the four groups for the selected MMPI-2-RF scales.

**Table 3:** Means and SD in the four experimental groups for *T*-scores in the selected MMPI-2-RF scales, with *post hoc* test results.

	H/U (*n* = 35) *M* (*SD*)	FG/U (*n* = 33) *M* (*SD*)	H/S (*n* = 33) *M* (*SD*)	FG/S (*n* = 34) *M* (*SD*)
**Validity scales**			
L-r	48.54 (8.33) A	64.21 (12.68) B	48.03 (7.10) A	70.50 (6.09) C
F-r	47.09 (11.39) A	42.36 (8.09) A, B	44.85 (11.70) A, B	39.12 (7.45) B
K-r	52.80 (4.70) A	57.58 (6.33) B	51.67 (6.56) A	62.12 (4.13) C
**RC scales**				
RCd Demoralization	52.57 (6.29) A	48.57 (9.08) A, B	52.39 (8.41) A	43.88 (9.49) B
RC1 Somatic Complaints	53.40 (12.79) A	50.72 (13.90) A	48.93 (9.43) A	46.65 (13.97) A
RC2 Low Positive Emotions	49.14 (8.15) A	48.39 (8.92) A	47.24 (8.77) A	44.50 (5.96) A
RC3 Cynicism	53.74 (9.26) A	51.03 (6.59) A	51.45 (7.22) A	51.15 (10.86) A
RC4 Antisocial Behavior	53.80 (8.90) A	46.79 (9.33) B, C	52.97 (10.39) A, B	44.26 (11.26) C
RC6 Ideas of Persecution	48.57 (6.16) A	44.85 (9.41) A, B	45.73 (7.62) A, B	41.12 (6.65) B
RC7 Dysfunctional Negative Emotion	52.74 (6.70) A	48.97 (6.59) A	49.82 (6.71) A, B	44.11 (9.38) B
RC8 Aberrant Experiences	45.28 (11.45) A	40.88 (10.12) A, B	43.45 (9.54) A, B	38.42 (6.18) B
RC9 Hypomanic Activation	53.86 (8.24) A, B	47.76 (11.32) A, C	54.70 (6.66) B	47.26 (9.68) C
Total RC *T*-score	463.11 (32.34) A	427.93 (35.27) B	446.70 (37.12) A, B	401.47 (43.94) C

*H, honest; U, un-speeded; FG, faking-good; S, speeded; L-r, Lie scale; K-r, Correction scale; F-r, Frequencies scale. For each line, different letters indicate a significant difference between columns.*

In the L-r and K-r scales, a *post hoc* test showed that the FG/S group obtained significantly higher *T*-scores than the other three groups. The FG/U group obtained the second highest values (significantly different from those of the other three groups), while both H groups (U and S) obtained similar results. It is interesting to underline that the *T*-scores of the L-r and K-r scales were in the normal range in the two H groups, while the FG/S group showed a very high range in the L-r scale and the FG/U group showed a moderately high range in the same scale. The FG/U group showed a tendentially high range in the K-r scale and the FG/S group showed a moderately high range in the same scale. In the F Scale, scores for the H/U (higher) and FG/S (lower) groups significantly differed.

In the RC scales, all scores were in the normal range. In RC1 and RC2, no significant differences were found between groups. Results showed the same trend, with the FG/S group achieving the lowest value, followed by the H/S, FG/U, and H/U groups. Similarly, no significant differences between groups were found in RC3, with the difference between all four groups bounded within 2.7 points. In RC4, RC6, RC7, and RC8, only the H/U (highest scores) and FG/S (lowest scores) groups differed markedly. In RCd and RC9, the H groups differed from the FG groups in the S condition. In the Total scale, the FG/S group reported lower scores than the other three groups.

### Subjective Psychological Being

In the two VAS, MANOVA results revealed a significant effect for honesty [Wilks’ lambda *F*(2,130) = 71.170; *p* < 0.001; $\eta_{\text{p}}^{2}$ = 0.523], speed [Wilk’ lambda *F*(2,130) = 45.257; *p* < 0.001; $\eta_{\text{p}}^{2}$ = 0.410], and the interaction between honesty and time [Wilks’ lambda *F*(2, 130) = 4.030; *p* = 0.020; $\eta_{\text{p}}^{2}$ = 0.058]. **Table 4** reports the descriptive values of the four groups for the VAS, with *post hoc* results.

**Table 4:** Means and SD in the four experimental groups for VAS, with *post hoc* test results.

VAS	H/U (*n* = 35) *M* (*SD*)	FG/U (*n* = 33) *M* (*SD*)	H/S (*n* = 33) *M* (*SD*)	FG/S (*n* = 34) *M* (*SD*)
Tension	2.83 (1.76) A	4.84 (1.86) B	3.76 (2.08) A, B	7.50 (1.78) C
Fatigue	3.54 (1.42) A	5.67 (1.31) B	5.61 (1.30) B	7.29 (1.40) C

*VAS, Visual Analog Scale; H, honest; U, un-speeded; FG, faking-good; S, speeded.*

A *post hoc* test revealed that tension was higher in the FG/S group. For fatigue, the H/U group was lowest while the FG/S group was highest. We also examined the correlation between the sum of the two VAS (VAS-T and VAS-F) and the RLs in the three validity scales. The results showed a positive correlation with the L-r scale (*rs* = 0.357; *p* < 0.01) and the K-r scale (*rs* = 0.426; *p* < 0.01). No significant correlation was found in the F-r scale (*rs* = 0.191).

### Regression Analyses

A test of the full model against a constant only model was statistically significant (**Table 5**), indicating that the set of predictors reliably distinguished between the presence or absence of honesty [χ^2^(6) = 121.075, *p* < 0.001]. Nagelkerke’s *R*^2^ of 0.790 indicated a moderately strong relationship between prediction and grouping. Prediction success, overall, was 93.3% (94.1% for the H condition and 92.5% for the FG condition). The Wald criterion demonstrated that three variables made a significant contribution to the prediction (first and second part of the inventory, K-r, and RL). The Exp(B) value indicated that when these three variables raised by one point, the possibility of faking-good behavior increased 0.28, 1.95, and 0.36, respectively.

**Table 5:** Binomial logistic regression.

Completion time	B	Exp(B)	Chi-squared Wald test	
			Test	*p*
First part	−1.243	0.288	16.843	<0.001
Second part	0.666	1.946	4.844	0.028
Third part	−0.113	0.893	0.380	NS
L-r	−0.438	0.646	0.998	NS
F-r	0.447	1.564	2.897	NS
K-r	−1.016	0.362	5.293	0.021

*L-r, Lie scale; K-r, Correction scale; F-r, Frequencies scale; NS, not significant.*

## Discussion

The principal aim of this study was to assess whether RL in a self-administered questionnaire could discriminate between honest and faking-good respondents, with particular attention given to the effect of time pressure (in a speeded condition) on faking-good. We were interested in gaining insight into this relationship in order to test the use of time as another variable of validity in self-reported inventories, particularly in cases where subjects could be motivated to represent themselves in a better light (e.g., personnel selection). While this topic has been researched since the 1970s, the results have been mixed.

Overall, our results found that H respondents were faster than FG ones. In more detail, our data confirmed H1 (relating to different completion times between groups). Briefly, there was a faster group (H/S) and a slower group (FG/U), and FG groups were always slower than H groups under the same speed conditions. H2 was also mostly confirmed by our data. We found a clear progression of completion times in the two H groups, while in both FG groups only the completion time of the third part was higher than that of the first two. H3 was partially confirmed. In the two scales of positive self-representation (L-r and K-r), FG groups registered longer completion times than H groups. The L-r scale produced a clearer result, differentiating between all four groups, while in K-r, FG groups took a similar time to respond, suggesting that the reasoning was complex and required more difficult choices. After all—with respect to the L-r scale—the K-r scale assesses more complex behaviors, concerning live adaptation and the ability to control one’s own reactions (Friedman et al., 2014). In the F-r scale, results were confused and did not confirm our hypothesis; this may have been due to the fact that we tested normal subjects (as discussed further, below).

However, the finding that emerged most clearly was the shorter completion times and RLs of H groups, relative to FG groups. How should this finding be explained? Over time, researchers have developed various interpretive models. Markus (1977) and Kuiper (1981) hold that schema-relevant characteristics are more difficult to determine than self-schema characteristics. According to Holden et al. (1992), the larger RL of fakers can be attributed to their greater use of cognitive processes relative to honest responders: dishonest respondents must evaluate schematic information before they choose not to provide self-schematic information. On the other hand, self-schematic information is sufficient for honest respondents, who answer more quickly. According to Vasilopoulos et al. (2000) the larger RL of fakers is produced by higher emotional arousal caused by the fear of detection. According to DePaulo et al. (2003), fakers take longer to respond because the schema of an ideal respondent is less accessible than the self-schema of an honest respondent. The present results relating to the L-r scale support Holden (1995) theory, as the L-r scale comprises 14 items (11 false and only 3 true). Similar to the findings of Holden et al. (1992), we found that FG groups took more time to respond to this scale, as it prevalently scores false. A similar interpretation applies to the K-r scale, which is composed of 16 items (14 false and only 2 true).

The specific pattern of RL and completion time found in our data suggest that, while H groups showed progressive fatigue over the full execution of the test, FG groups’ fatigue was interpolated with a longer latency, probably due to the effort required to provide good (and perhaps false) self-information. In the first part of the questionnaire, FG groups reported slower completion times, probably because they were learning a model of FG. In other words: (a) FG respondents may have taken more time to fill in the different sections of the questionnaire than H respondents because they needed more time to think before answering; (b) the natural effect of fatigue in FG respondents may have been amplified by an initial difficulty in learning the FG response model, and this may have increased the completion time in the first part of the test; and (c) the influence of tension and anxiety may have muddled FG respondents’ thoughts. The data underlines that the mental task and cognitive process of FG respondents were more complicated than those of H respondents.

The results of the MMPI-2-RF validity scales support H4. FG groups reported higher values on the positive self-presentation scales (L-r and K-r), as also found by others (e.g., Brunetti et al., 1998). The F-r data were complex and only partially satisfied H4. We believe that this occurred because we tested a normal sample, and thus the “floor effect” described by Peterson et al. (1989) was high (honest respondents endorsed so few psychopathology-related items that, when asked to fake good, few differences could be noted). The results also stressed that speed induced FGs to significantly improve their self-representation in the L-r and K-r scales. This is an interesting outcome, which we attributed to the S condition leading respondents to drastically reduce their consideration of the appropriateness of lying on items about a virtuous attitude. In H subjects, however, speed did not produce differences in L-r, F-r, and K-r scores; this suggests that answering honestly at speed does not lessen scores relative to answering at leisure. The data thus confirm the work of Khorramdel and Kubinger (2006), who found that faking in responding to dichotomous items was accentuated under time pressure. Scores of the RC scales did not reach clinical significance. However, this outcome should take into account the fact that the sample did not belong to a clinical population.

With respect to the variables of tension during the trial and fatigue after the trial (H5), the FG/S group achieved the highest scores, followed by the FG/U and H/S groups. It seems that both the fake good request and the speed request required additional psychological effort on the part of respondents. In other words, the H/S group had to think only about being fast, the FG/U group had to think about only reflecting themselves in the best light, while the FG/S group faced both challenges: going fast and showing their best face. Our results substantiate previous data (see McDaniel and Timm, 1990) showing increased emotional arousal experienced by subjects making an impression managed response under time restriction (Temple and Geisinger, 1990).

With regard to H6, increased completion time in the first part and the K-r scale decreased the probability of honest responding; in contrast, increased completion time in the central part of the test increased the probability of honest responding. These results align with our previous interpretations: in the first part, FG respondents had to learn a schema of dishonesty, and so longer completion times in this section could lead us to believe that subjects were fakers. Further, the K-r scale required complex answers, and thus a long RL may have been associated with malingering behaviors. Moreover, if completion of the second part did not increase significantly relative to the first, there was a greater possibility of dishonest responding.

In conclusion, our data were consistent with the findings of McDaniel and Timm (1990), Walczyk et al. (2005), Foerster et al. (2013), and Maricuţoiu and Sârbescu (2016), which point to an increased response time among FG groups. Moreover, the S condition might more accurately enable the detection of dishonesty, as also found by Khorramdel and Kubinger (2006) and Shalvi et al. (2013), using other questionnaires.

### Strengths and Limitations

The present study adds useful insight to the debate over the response times of fakers, while examining variables that have not yet been considered in the literature (e.g., completion times for individual sections of a questionnaire). Furthermore, to the best of our knowledge, this study was the first to jointly evaluate honesty conditions and time pressure in the MMPI-2-RF.

Nevertheless, there are two important limitations of this study that require additional research to overcome: (a) the analyzed group was selected for specificity (graduate males aged 25–30 years), and this reduced the generalizability of the findings; and (b) the sample size was small. Moreover, in the future, it would be useful to study a sample of subjects in an ecological condition (e.g., psycho-aptitude or forensic evaluation) and to examine RL differences according to item content. Future studies could investigate whether RLs are associated with particular scales of personality inventories within specific assessment settings in which malingerers must fake good to achieve certain goals.

## Conclusion

The results suggest that, in computerized self-administered personality and psychopathology tests, RL and completion times could be usefully treated as additional indexes of falsification in self-representation. Furthermore, as speed increases our ability to identify falsifying subjects, time conditions could be applied to selection contexts in which self-reports are often used.

## Ethics Statement

This study was carried out with written informed consent by all subjects and was approved by the local ethics committee (Board of the Department of Human Neuroscience, Faculty of Medicine and Dentistry, Sapienza University of Rome).

## Author Contributions

All authors helped to conceive and plan the study and prepared and approved the final manuscript. PR conducted the data collection and produced the first draft of the final manuscript. SF, MV, and DM supervised the data collection. PR and CM conducted the analyses and wrote the manuscript. MV and DM carefully read the final version of the manuscript.

## Conflict of Interest Statement

The authors declare that the research was conducted in the absence of any commercial or financial relationships that could be construed as a potential conflict of interest. The handling Editor declared a past co-authorship with two of the authors PR and SF.

## References

[B1] Anastasi A. (1988). *Psychological Testing*, 6th Edn. New York, NY: Macmillan.

[B2] Anderson J. L. Sellbom M. Pymont C. Smid W. De Saeger H. Kamphuis J. H. (2015). Measurement of DSM-5 section II personality disorder constructs using the MMPI-2-RF in clinical and forensic samples. *Psychol. Assess.* 27 786–800. 10.1037/pas0000096 25799092

[B3] Ben-Porath Y. S. Tellegen A. (2008). Empirical correlates of the MMPI-2 restructured clinical (RC) scales in mental health, forensic, and nonclinical settings: an introduction. *J. Pers. Assess.* 90 119–121. 10.1080/00223890701845120 18444104

[B4] Ben-Porath Y. S. Tellegen A. (2008/2011). *MMPI-2-RF (Minnesota Multiphasic Personality Inventory-2 Restructured Form) Manual for Administration, Scoring, And Interpretation.* Minneapolis, MN: University of Minnesota Press.

[B5] Brunetti D. G. Schlottmann R. S. Scott A. B. Hollrah J. L. (1998). Instructed faking and MMPI-2 response latencies: the potential for assessing response validity. *J. Clin. Psychol.* 54 143–153. 10.1002/(SICI)1097-4679(199802)54:2<143::AID-JCLP3>3.0.CO;2-T 9467758

[B6] Butcher J. N. Graham J. R. Ben-Porath Y. S. Tellegen A. Dahstrom W. G. Kaemmer B. (2001). *MMPI-2. Manual for Administration and Scoring.* Minneapolis, MN: University of Minnesota Press.

[B7] Costa P. T. McCrae R. R. (1992). *NEO PI-R Professional Manual: Revised NEO Personality Inventory (NEO-PI-R) and NEO Five-Factory Inventory (NEO-FFI).* Odessa, FL: Psychological Assessment Resources.

[B8] DePaulo B. M. Lindsay J. J. Malone B. E. Muhlenbruck L. Charlton K. Harris C. (2003). Cues to deception. *Psychol. Bull.* 129 74–112. 10.1037/0033-2909.129.1.74 12555795

[B9] Dunn T. G. Lushene R. E. O’Neil H. F. (1972). Complete automation of the MMPI and a study of its response latencies. *J. Consult. Clin. Psychol.* 39 381–387. 10.1037/h0033855 4405459

[B10] Fluckinger C. D. McDaniel M. A. Whetzel D. L. (2008). “Review of faking in personnel selection,” in *In Search of the Right Personnel*, ed. Mandal M. (New Delhi: McMillian).

[B11] Foerster A. Pfister R. Schmidts C. Dignath D. Kunde W. (2013). Honesty saves time (and justifications). *Front. Psychol.* 4:473. 10.3389/fpsyg.2013.00473 23888151 PMC3719030

[B12] Friedman A. F. Bolinskey P. K. Levak R. W. Nichols D. S. (2014). *Psychological Assessment with the MMPI-2/MMPI-2-RF.* New York, NY: Routledge.

[B13] Hathaway S. R. McKinley J. C. (1951). *Minnesota Multiphasic Personality Inventory.* Minneapolis, MN: University of Minnesota Press.

[B14] Hathaway S. R. McKinley J. C. (1989). *Minnesota Multiphasic Personality Inventory – 2.* Minneapolis, MN: University of Minnesota Press.

[B15] Holden R. R. (1995). Response latency detection of fakers on personnel tests. *Can. J. Behav. Sci.* 27 343–355. 10.1037/0008-400X.27.3.343

[B16] Holden R. R. Kroner D. G. (1992). Relative efficacy of differential response latencies for detecting faking on a self-report measure of psychopathology. *Psychol. Assess.* 4 170–173. 10.1037/1040-3590.4.2.170

[B17] Holden R. R. Kroner D. G. Fekken G. C. Popham S. M. (1992). A model of personality test item response dissimulation. *J. Pers. Soc. Psychol.* 63 272–279. 10.1037/0022-3514.63.2.272 11474721

[B18] Holden R. R. Lambert C. E. (2015). Response latencies are alive and well for identifying fakers on a self-report personality inventory: a reconsideration of van Hooft and Born (2012). *Behav. Res. Methods* 47 1436–1442. 10.3758/s13428-014-0524-5 25381021

[B19] Holden R. R. Wood L. L. Tomashewski L. (2001). Do response time limitations counteract the effect of faking on personality inventory validity. *J. Pers. Soc. Psychol.* 81 160–169. 10.1037/0022-3514.81.1.160 11474721

[B20] Hsu L. M. Santelli J. E. Hsu J. R. (1989). Faking detection validity and incremental validity of response latencies to MMPI subtle and obvious items. *J. Pers. Assess.* 53 278–295. 10.1207/s15327752jpa5302_6

[B21] Khorramdel L. Kubinger K. D. (2006). The effect of speediness on personality questionnaires: an experiment on applicants within a job recruiting procedure. *Psychol. Sci. Q.* 48 378–397.

[B22] Kuiper N. A. (1981). Convergent evidence for the self as a prototype: the “inverted-U RT effect” for self and other judgments. *Pers. Soc. Psychol. Bull.* 7 438–443. 10.1177/014616728173012

[B23] Kuncel R. B. (1973). Response processes and relative location of subject and item. *Educ. Psychol. Meas.* 33 545–563. 10.1177/001316447303300302

[B24] Maricuţoiu L. P. Sârbescu P. (2016). The relationship between faking and response latencies: a meta-analysis. *Eur. J. Psychol. Assess.* 1–11. 10.1027/1015-5759/a000361

[B25] Markus H. (1977). Self-schemata and processing information about the self. *J. Pers. Soc. Psychol.* 35 63–78. 10.1037/0022-3514.35.2.63

[B26] McCormack H. M. Horne D. J. Sheather S. (1988). Clinical applications of visual analogue scales: a critical review. *Psychol. Med.* 18 1007–1019. 10.1017/S0033291700009934 3078045

[B27] McDaniel M. A. Timm T. (1990). “Lying takes time: predicting deception in biodata using response latency,” in *Symposium Presented at the 98th Annual Conference of the American Psychological Association*, Boston, MA.

[B28] Nowakowska M. (1970). A model of answering to a questionnaire item. *Acta Psychol.* 34 420–439. 10.1016/0001-6918(70)90036-3

[B29] Peterson G. W. Clark D. A. Bennett B. (1989). The utility of MMPI subtle, obvious scales for detecting fake good and fake bad response sets. *J. Clin. Psychol.* 45 575–582. 10.1002/1097-4679(198907)45:4<575::AID-JCLP2270450412>3.0.CO;2-F 2768496

[B30] Pierce C. A. Block R. A. Aguinis H. (2004). Cautionary note on reporting eta-squared values from multifactor ANOVA designs. *Educ. Psychol. Meas.* 64 916–924. 10.1177/0013164404264848

[B31] Rogers T. B. (1971). The process of responding to personality items: some issues, a theory, and some research. *Multivariate Behav. Res. Monogr.* 6 1–65.

[B32] Rogers T. B. (1977). Self-reference in memory: recognition of personality items. *J. Res. Pers.* 11 295–305. 10.1016/0092-6566(77)90038-1

[B33] Scheffè H. (1959). *The Analysis of Variance.* New York, NY: John Wiley & Sons.

[B34] Shalvi S. Eldar O. Bereby-Meyer Y. (2013). Honesty requires time: a reply to Foerster (2013). *Front. Psychol.* 4:634. 10.3389/fpsyg.2013.00634 24133466 PMC3783836

[B35] Sirigatti S. Faravelli C. (2012). *MMPI-2 RF: Adattamento Italiano. Taratura, Proprietà Psicometriche e Correlati Empirici.* Florence: Giunti O.S. Organizzazioni Speciali.

[B36] Tarescavage A. M. Corey D. M. Gupton H. M. Ben-Porath Y. S. (2015). Criterion validity and practical utility of the Minnesota multiphasic personality inventory–2–restructured form (MMPI–2–RF) in assessments of police officer candidates. *J. Pers. Assess.* 97 382–394. 10.1080/00223891.2014.995800 25588076

[B37] Tellegen A. Ben-Porath Y. S. (1992). The new uniform T scores for the MMPI-2: rationale, derivation, and appraisal. *Psychol. Assess.* 4 145–155. 10.1037/1040-3590.4.2.145

[B38] Tellegen A. Ben-Porath Y. S. McNulty J. L. Arbisi P. A. Graham J. R. Kaemmer B. (2003). *RC Scales Test Monograph.* Minneapolis, MN: University of Minnesota Press.

[B39] Temple D. E. Geisinger K. F. (1990). Response latency to computer-administered inventory items as an indicator of emotional arousal. *J. Pers. Assess.* 54 289–297. 10.1207/s15327752jpa5401&2_27

[B40] Van Hooft E. A. J. Born M. P. (2012). Intentional response distortion on personality tests: using eye-tracking to understand response processes when faking. *J. Appl. Psychol.* 97 301–316. 10.1037/a0025711 21967296

[B41] Vasilopoulos N. L. Reilly R. R. Leaman J. A. (2000). The influence of job familiarity and impression management on self-report measure scale score and response latencies. *J. Appl. Psychol.* 85 50–64. 10.1037/0021-9010.85.1.50 10740956

[B42] Walczyk J. J. Roper K. S. Seemann E. Humphrey A. M. (2003). Cognitive mechanisms underlying lying to questions: response time as a cue to reception. *Appl. Cogn. Psychol.* 17 755–774. 10.1002/acp.914

[B43] Walczyk J. J. Schwartz J. P. Clifton R. Barett A. Wei M. Zha P. (2005). Lying person to person about life events: a cognitive framework for lie detection. *Pers. Psychol.* 58 141–170. 10.1111/j.1744-6570.2005.00484.x

[B44] Ziegler M. MacCann C. Roberts R. D. (eds). (2012). “Faking: knowns, unknowns, and points of contention,” in *New Perspectives on Faking in Personality Assessment* (New York, NY: Oxford University Press), 3–16.

